# Clinical efficacy and influence on the quality of life of children with precocious puberty treated by combination of cluster nursing and triptorelin

**DOI:** 10.1097/MD.0000000000044261

**Published:** 2025-09-19

**Authors:** Hui Wang, Juan Chen

**Affiliations:** a Department of Outpatient, The Fourth Affiliated Hospital of Soochow University (Suzhou Dushu Lake Hospital), Suzhou, China.

**Keywords:** clinical effect, cluster nursing, music score Rui Lin, precocious puberty in children, quality of life

## Abstract

This study evaluates clinical efficacy of cluster nursing plus triptorelin in pediatric precocious puberty and its impact on patient quality of life. A total of 110 children with precocious puberty hospitalized between June 2022 and June 2024 were enrolled. To ensure comparability, participants were grouped using propensity score matching based on the nursing method previously received, along with sex, age, and baseline characteristics. The subjects were then randomized into 2 groups: the control group (n = 55), which received standard therapy, and the study group (n = 55), which received cluster nursing plus triptorelin treatment in addition to the standard care regimen. The clinical efficacy, growth and development indicators, children’s behavioral problems, quality of life, and the occurrence of adverse reactions were observed and compared between the 2 groups. The total effective rate after intervention was significantly higher in the study group (96.36%) than in the control group (85.45%) (*P* < .05). After intervention, bone age and predicted adult lifetime high level were increased in both groups compared with before intervention, luteinizing hormone and T were decreased compared with before intervention, the study group demonstrated significantly higher predicted adult height versus controls, while bone age, luteinizing hormone, and T levels were significantly lower versus controls (*P* < .05). Post-intervention depression scores, social withdrawal, hyperactivity and aggression in both groups were lower than before intervention, and the scores of depression, social withdrawal, hyperactivity, and aggression in the study group were lower than those in the control group (*P* < .05). After intervention, scores of family life, peer interaction, school life, living environment, self-knowledge, physical emotion, living environment, anxiety experience, and depression experience in both groups were higher than before intervention, and scores of family life, peer interaction, school life, living environment, self-knowledge, physical emotion, anxiety, and depression scores significantly exceeded control group levels in the study group (*P* < .05). Post-intervention adverse reaction incidence was 3.64% lower in the study group versus controls (10.91%) (*P* < .05). The combination of cluster nursing and triptorelin therapy can effectively control the growth and development of children with precocious puberty, improve their behavior problems and quality of life. It has high safety and effectiveness and is worthy of popularization and application.

## 1. Introduction

Precocious puberty is an endocrine disease caused by abnormal growth and development in children. Epidemiological studies^[1]^ demonstrate that precocious puberty affects approximately 0.6% of children between ages 6 to 18 in China. Female prevalence (0.48%) exceeds male prevalence (0.38%), indicating a gender-based disparity. These statistics suggest that over 530,000 Chinese children experience this condition nationwide. With the changes in lifestyle, the incidence rate of this disease is increasing year by year, seriously affecting the normal development of children. Children with precocious puberty are children with abnormal sexual development who are clinically characterized by premature sexual maturity. Clinical manifestations typically include premature emergence of secondary sexual traits prior to standard developmental timelines. Boys mainly show tall, muscular, and muscular symptoms before the age of 9. Protruding Adam apple, thick hair, enlarged testicles and penis, development of pubic hair, etc. Girls usually show increased subcutaneous fat, delicate skin, breast development, wide pelvis, appearance of armpit hair, and pubic hair.^[2,3]^ The cause of the disease is complex, and its pathological mechanism is divided into central sexual maturation and peripheral sexual maturation. Central sexual maturation has the same programmed process of normal development and maturation of sex hormones as normal pubertal development, but peripheral sexual maturation does not. In this process, both of them have early appearance of secondary sexual characteristics.^[4,5]^ Due to the large differences in pathological mechanisms, clinical treatment options are often given according to the pathological mechanisms. Among them, peripheral sexual maturity is usually treated with surgery and cortisol replacement therapy, while children with central sexual maturity are mostly given gonadotropin-releasing hormone (GnRH) analogue treatment to inhibit GnRH expression and alleviate the sexual development process.^[6,7]^ As a commonly used GnRH analog, metroprelin can effectively bind to GnRH receptors, control the process of sexual development, and has good therapeutic effects.^[8]^

Some scholars have pointed out^[9]^ that due to the development of internal and external genitalia and the emergence of secondary sexual characteristics, children with precocious puberty are prone to low self-esteem, social isolation, and early sexual behavior. Compared with their peers, children with precocious puberty are more likely to suffer from depression and have poor cognitive abilities in children. Limited ability makes it difficult to follow doctor’s instructions and take medication, which elevates challenges in disease progression control and nursing care, while weakening treatment outcomes.Consequently, identifying effective therapeutic strategies holds substantial importance for enhancing disease understanding and optimizing clinical outcomes. Domestic and foreign reports mostly focus on the prevention, control and treatment of precocious puberty, and there are few studies on the nursing management of children.^[10,11]^ A total of 110 children diagnosed with precocious puberty were recruited from our institution during the period of June 2020 to June 2022 to evaluate clinical outcomes and growth metrics associated with comprehensive care combined with triptorelin therapy. Children’s behavioral problems, quality of life, occurrence of adverse reactions, etc., in order to provide scientific basis for clinical disease treatment and nursing plan selection.

## 2. Materials and methods

### 2.1. General information

This retrospective study was approved by the Ethics Committee of The Fourth Affiliated Hospital of Soochow University (Suzhou Dushu Lake Hospital). It enrolled 110 children with precocious puberty who were hospitalized between June 2022 and June 2024. To ensure comparability, subjects were grouped using propensity score matching based on the nursing method previously received, as well as sex, age, and baseline characteristics. Subjects were then randomized into control and experimental cohorts (n = 55 per group). The control group received routine nursing and medication, while the research group additionally received cluster nursing and triptorelin treatment alongside the standard care protocol. No significant between-group differences existed (*P* > .05), ensuring comparability. Ethical clearance was secured from the institutional review committee.

### 2.2. Inclusion and exclusion criteria

Inclusion criteria: fulfilled pediatric precocious puberty diagnostic standards outlined in the “Expert Consensus on Diagnosis and Treatment of Central Precocious Puberty (2022)”^[12]^; diagnosed as precocious puberty in children by pelvic B-ultrasound, bone age, and sex hormone examination; age ≤ 12 years old; the volume of the uterus and ovary of girls is >1 mL, there are multiple follicles with a diameter of ≥4 mm, the GnRH stimulation test is positive, and the luteinizing hormone (LH)/follicle stimulating hormone (FSH) > 0.6^[13]^; the boys have enlarged testicles, thickened penis, and growth of pubic hair; no cognitive impairment or mental disorder; the research subjects and relatives are informed of the research content, and voluntarily signed the informed consent form.

Exclusion criteria: those without serious lesions of important organs such as heart, liver, kidney, etc; precocious puberty induced by non-increased GnRH; those with central nervous system diseases and malignant tumors; those with epinephrine, thyroid, ovary, etc. Those with diseases; previous drug treatment history; those with missing clinical data, drug allergies, and poor compliance.

### 2.3. Research methods

Standard care and conventional pharmacotherapy were administered to the control cohort: routine nursing: after admission, all children will establish personal case files, including gender, age, medication history, allergy history, drug treatment and dosage, follow-up time, etc, and inform them of compliance medication prescribed by the doctor and regular review; provide health education related to precocious puberty to patients and their families, including the causes of the disease, treatment methods, prognosis, precautions, and complications; inform them to pay attention to the child’s lifestyle, diet, nutrition, and psychological state, and provide encouragement and support for children. Drug treatment: give GnRH analogues and vitamins according to the pathogenesis factors.

The research group additionally received cluster nursing combined with qurelin treatment alongside the control protocol: *cluster nursing*: a clustered nursing team composed of pediatricians, senior head nurses, responsible nurses, psychological counselors, etc. Nursing staff carry out special training on theoretical knowledge and practical skills focusing on the care of children with precocious puberty. Only those who pass the training can participate in the clustered care of children with clinical precocious puberty. Development of nursing intervention plan: review relevant literature on the care of children with precocious puberty, summarize the overall nursing plan for children with precocious puberty based on clinical experience, and then formulate a targeted cluster nursing plan based on the child’s disease progress, family situation, etc. Health education and psychological intervention: after admission, communicate with the children and their families in a friendly and kind manner, fully understand the children’s condition, family situation, disease cognition, and psychological state, communicate with the children through a positive attitude, and establish a good relationship with the children. Trust in medical staff and their work, use the things that the children are interested in as the starting point, and understand the children’s personality, hobbies, and disease cognition through concise conversations to avoid causing the children to be impatient and resistant. According to the education level and acceptable methods of the children and their families, inform the children and their families about the mechanism, treatment plan, precautions, etc, of precocious puberty, inform the children to pay attention to menstrual hygiene care, etc, and pay full attention to the problem of precocious puberty; through communication with the children and their families, communicate with their family members, understand the psychological state of the children, explain to them that growth and development are signs of maturity, cause them to correctly view physical changes, establish a correct understanding of sexual knowledge, actively and bravely face past events, and ensure their physical and mental health development; establish another line online consultation platform provides a private communication platform for children and their families, strengthens communication between medical staff, parents and children, promptly assesses and provides family care, lifestyle and disease intervention, etc; informs parents to establish correct sexual concepts, comforts and encourage children with precocious puberty to correctly view the problem, help them actively participate in offline knowledge training and social group activities, and promote physical and mental health. Lifestyle intervention: instruct patients to assist children in regular and quantitative medication use, regular review, and avoid increasing or reducing drug dosage or frequency of administration on their own. If adverse events occur, medical staff must be informed promptly and targeted treatment must be given; provide a reasonable diet for nutritional status and physical condition, eat more seasonal fresh vegetables and fruits, supplement vitamins, avoid intake of soy products, dairy products, honey, etc., avoid intake of fried foods, desserts, drinks and other foods, and pay attention to a diversified diet. Ensure balanced nutrition; provide exercise programs according to their physical condition, encourage them to exercise step by step, improve the body’s immunity, and maintain a good figure; develop a good daily routine and ensure sleep quality. *Triperelin (produced by Ipsen Pharma Biotech, Signes, Provence-Alpes-Côte d'Azur, France; registration No. H20140298*) *treatment*: the initial dose was given subcutaneously according to body weight, including 1.875 mg/ time with body weight < 20 kg; the body weight is 20 to 30 kg, given 2.5 mg/ time; patients with body weight > 30 kg were given 3.75 mg/time, once every 28 days, and the dose was adjusted according to height, weight and gonadal inhibition, with the dose adjustment range ranging from 0.06 to 0.10 mg/kg, once every 28 to 35 days, and continued treatment for 6 months.

### 2.4. Observation indicators

Clinical efficacy: in accordance with the treatment efficacy evaluation standards. Markedly effective: the growth rate returned to normal after treatment, the bilateral mammary nucleus tissue retracted > 50%, and the development of the uterus and ovaries basically stagnated; effective: the growth rate after treatment was significantly lower than before treatment, and the bilateral mammary nucleus tissue retracted within a range of 30% to 50%, the development of the uterus and ovaries is significantly slow; invalid: the growth rate does not change or accelerates after treatment, the bilateral mammary nucleus tissue does not shrink significantly or continues to increase, and the development of the uterus and ovaries does not change or continues to develop. Growth and development indicators: anteroposterior X-rays were used to examine the wrist joints before intervention and 6 months after intervention, and the bone age was calculated based on the growth status of wrist joint bones, the formation of ossification centers, and living age; the Bayley-Pinneau method was used to calculate the bone age of the children.^[14]^ The standard deviation unit curve chart of height and weight predicts the predicted adult lifetime height of children before intervention and 6 months after intervention. Before and 6 months after intervention, 3 mL fasting venous blood was drawn, then centrifuged at 3000 r/min for 15 minutes.The upper serum was taken, and the levels of LH and testosterone (T) were measured using a chemiluminescence immunoassay analyzer. Assessment of children’s behavioral problems: The Achenbach Child Behavior Scale^[15]^ was used to assess children’s behavioral problems before the intervention and 6 months after the intervention. The scale includes factors such as depression, social withdrawal, hyperactivity, and aggression, the scale is based on a 0 to 2-level scoring system, with 0 indicating no problem, 1 indicating mildly present, and 2 indicating definitely present. Quality of life: life satisfaction was assessed using the inventory of subjective life quality^[16]^ for pediatric populations at baseline and 6-month posttreatment. This scale includes cognitive components and emotional components, among which cognitive components The components include 5 dimensions: family life, peer interaction, school life, living environment, and self-understanding. The emotional components include 3 dimensions: physical emotion, anxiety experience, and depression experience.Percent points were utilized for each dimension, with higher scores indicating, the higher the score. High indicates better quality of life and higher life satisfaction. Adverse reactions: the occurrence of adverse events such as nausea and vomiting was recorded, dizziness, slowed growth rate, poor bone age control, etc during the follow-up period.

### 2.5. Statistical processing

Statistical analyses used SPSS 24.0 (IBM Corporation, Armonk). Normally distributed data presented as mean ± standard deviation (±s) with intergroup comparisons by independent *t* test. Categorical data reported as n (%) analyzed by *χ*² test. Statistical significance defined as *P* < .05.

## 3. Results

### 3.1. Comparison of basic information

Baseline demographic and clinical characteristics including sex, age, illness duration, BMI, height, weight, and Tanner staging demonstrated no significant between-group disparities (*P* > .05). See Table 1.

**Table 1:** Comparison of basic data between the 2 groups.

Group		Control group (n = 55)	Experiment group (n = 55)	*t*	*P*
Gender (n)	Male	14 (25.45)	16 (29.09)	5.236	.360
	Female	41 (74.55)	39 (70.91)		
Age		7.36 ± 1.48	7.40 ± 1.49	5.706	.095
Course of disease (months)		5.36 ± 1.18	5.34 ± 1.17	7.132	.180
BMI (kg/m^2^)		16.35 ± 1.65	16.38 ± 1.64	5.948	1.035
Height (cm)		132.68 ± 5.62	134.69 ± 5.06	3.956	.840
Weight (kg)		29.05 ± 5.76	29.09 ± 5.72	5.623	.746
Tanner staging (n)	II grade	17 (30.91)	17 (30.91)		
	III grade	26 (47.27)	27 (49.09)		
	IV grade	12 (21.82)	11 (20.00)		

### 3.2. Comparison of clinical efficacy

Results showed the research group’s total effective rate post-intervention (96.36%) was significantly higher than the control group’s (85.45%), with a statistically significant difference(*P* < .05). See Table 2.

**Table 2:** Comparison of clinical efficacy between the 2 groups (cases, %).

Group	Control group (n = 55)	Experiment group (n = 55)	*χ*2	*P*
Effective	23 (41.82)	41 (74.55)	–	–
Efficient	24 (43.64)	12 (21.82)	–	–
Invalid	8 (14.55)	2 (3.64)	–	–
Total effective rate	85.45%	96.36%	5.623	.192

### 3.3. Comparison of growth and development indicators

Baseline comparisons showed no statistically significant differences in bone age, predicted adult height, LH, or T levels between groups (*P* > .05). Post-intervention measurements revealed increased bone age and predicted adult height in both groups compared to baseline, whereas LH and T levels decreased in both groups versus pretreatment values. The study group’s predicted adult lifelong high levels were higher than those of the control group, and bone age, LH, while T levels were markedly reduced compared to the control group, showing statistical significance (*P* < .05). Refer to Table 3 and Figure 1.

**Table 3:** Comparison of growth and development indexes between the 2 groups ($\bar{x}\pm \;s$).

Group	Time	Control group (n = 55)	Experiment group (n = 55)
Bone age (yr)	Before intervention	7.85 ± 0.62	7.89 ± 0.58
	After intervention	8.59 ± 0.71*	8.01 ± 0.67*^,^#
Predicting lifetime height in adulthood (cm)	Before intervention	154.60 ± 5.36	154.18 ± 5.38
	After intervention	156.97 ± 5.48*	159.62 ± 5.63*^,^#
LH (U/L)	Before intervention	3.86 ± 0.95	3.87 ± 0.94
	After intervention	2.79 ± 0.61*	1.25 ± 0.36*^,^#
T (nmol/L)	Before intervention	6.54 ± 1.87	6.58 ± 1.83
	After intervention	4.61 ± 1.36*	3.18 ± 1.09*^,^#

* Compared with before intervention, *P* < .05.

# Compared with the control group, *P* < .05.

**Figure 1.**
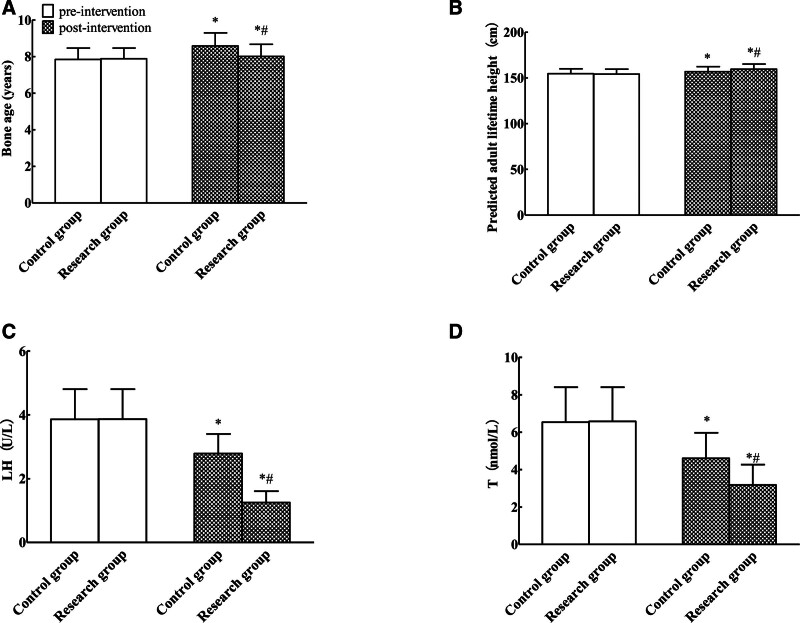
Comparison of growth and development indexes between the 2 groups (A: bone age; B: predicted adult lifetime height; C: LH; D: T, compared with before intervention, *P < .05; compared with the control group, ^#^*P* < .05). LH = luteinizing hormone.

### 3.4. Comparison of children’s behavioral problems

Achenbach scale scores were comparable between groups at baseline (*P* > .05). All behavioral domains (depression, social withdrawal, hyperactivity, and aggression) decreased post-intervention in both cohorts. The research group demonstrated significantly greater reductions in all scores versus controls, with statistically significant differences. (*P* < .05). See Table 4 and Figure 2.

**Table 4:** Comparison of children behavior problems between the 2 groups ($\bar{x}\pm \;s$, fraction).

Group	Time	Control group (n = 55)	Experiment group (n = 55)
Depression social withdrawal	Before intervention	10.35 ± 2.94	10.36 ± 2.98
	After intervention	7.58 ± 2.27*	5.64 ± 1.85*,#
Hyperactivity	Before intervention	11.60 ± 2.84	11.65 ± 2.85
	After intervention	8.37 ± 2.58*	5.37 ± 1.66*,#
Depression social withdrawal	Before intervention	11.57 ± 2.69	11.56 ± 2.71
	After intervention	8.65 ± 2.64*	6.37 ± 1.60*,#
Hyperactivity	Before intervention	13.95 ± 3.70	13.98 ± 3.67
	After intervention	9.68 ± 3.27*	6.35 ± 2.05*,#

* Compared with before intervention, *P* < .05.

# Compared with the control group, *P* < .05.

**Figure 2.**
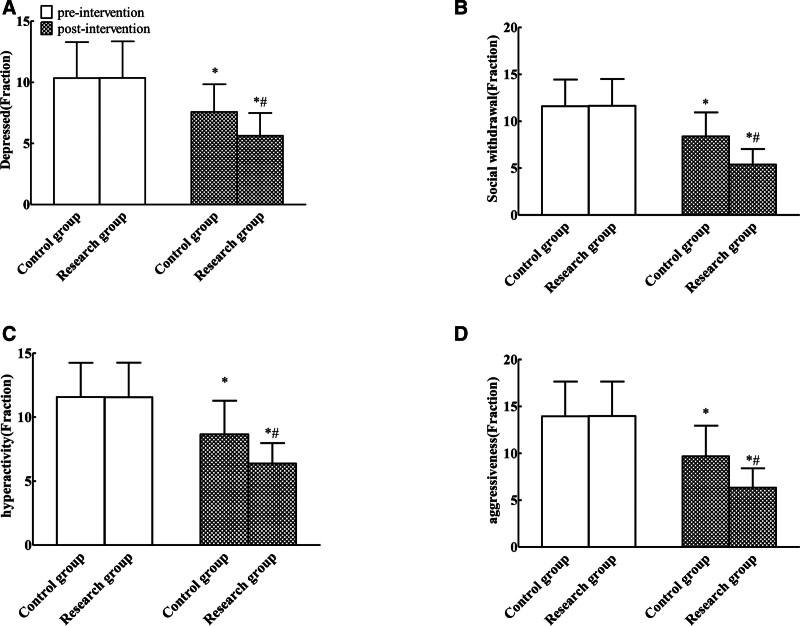
Comparison of children behavior problems between the 2 groups (A: depressed; B: social withdrawal; C: hyperactivity; D: aggressiveness, compared with before intervention, *P < .05; compared with the control group, ^#^*P* < .05).

### 3.5. Comparison of quality of life

Both patient groups demonstrated comparable pre-intervention inventory of subjective life quality dimension scores (*P* > .05), while post-intervention significant differences were observed in family life, peer interaction, and school life, living environment, self-understanding, physical emotion, and anxiety. The scores of experience and depressive experience were both higher than before the intervention. The scores of family life, peer interaction, school life, living environment, self-understanding, somatic emotion, anxiety experience, significantly increased depressive experience observed in the study group compared to controls held statistical and scientific significance (*P* < .05). See Table 5 and Figure 3.

**Table 5:** Comparison of quality of life between the 2 groups ($\bar{x}\pm \;s$, fraction).

Group	Time	Control group (n = 55)	Experiment group (n = 55)
Family life peer interaction	Before intervention	5.57 ± 0.69	5.59 ± 0.71
	After intervention	7.34 ± 0.80*	8.26 ± 1.68*^,^#
School life living environment	Before intervention	4.76 ± 0.55	4.73 ± 0.57
	After intervention	6.87 ± 0.72*	7.38 ± 0.79*^,^#
Self-knowledge somatic emotion	Before intervention	4.60 ± 0.56	4.60 ± 0.58
	After intervention	6.18 ± 0.71*	6.98 ± 0.89*^,^#
Anxiety experience	Before intervention	5.08 ± 0.62	5.06 ± 0.68
	After intervention	7.62 ± 1.09*	7.99 ± 1.16*^,^#
Family life peer interaction	Before intervention	5.38 ± 0.61	5.38 ± 0.67
	After intervention	7.06 ± 0.79*	7.63 ± 0.82*^,^#
School life living environment	Before intervention	5.63 ± 0.60	5.64 ± 0.62
	After intervention	6.92 ± 0.81*	7.38 ± 0.94*^,^#
Self-knowledge somatic emotion	Before intervention	5.94 ± 0.68	5.98 ± 0.67
	After intervention	6.08 ± 0.77*	6.59 ± 0.80*^,^#
Anxiety experience	Before intervention	5.60 ± 0.64	5.57 ± 0.68
	After intervention	7.31 ± 1.28*	7.89 ± 1.51*^,^#

* Compared with before intervention, *P* < .05.

# Compared with the control group, *P* < .05.

**Figure 3.**
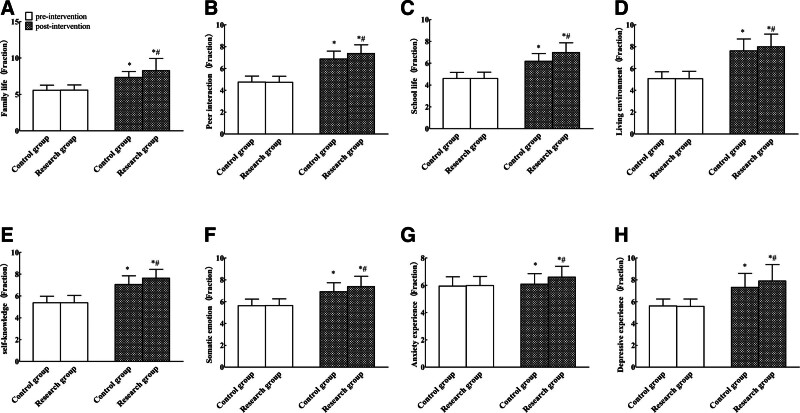
Comparison of quality of life between the 2 groups (A: family life; B: peer interaction; C: school life; D: living environment; E: self-knowledge; F: somatic emotion; G: anxiety experience; H: depressive experience, compared with before intervention, *P < .05; compared with the control group, ^#^*P* < .05).

### 3.6. Comparison of adverse reactions

Post-intervention adverse reaction incidence was 3.64% in the study group versus 10.91% in the control group, with a statistically significant difference(*P* < .05). See Table 6.

**Table 6:** Comparison of the incidence of adverse reactions between the 2 groups (cases, %).

Group	Control group (n = 55)	Experiment group (n = 55)	*χ*2	*P*
Feel sick and vomit	2 (3.64)	1 (1.82)	–	–
Dizziness	0 (0.00)	0 (0.00)	–	–
Growth rate slowed	1 (1.82)	0 (0.00)	–	–
Poor bone age control	3 (5.45)	1 (1.82)	–	–
Incidence of adverse reactions	10.91%	3.64%	5.095	.012

## 4. Discussion

Defined by premature genitalia and secondary sexual characteristic development (pre-age 9 in boys; pre-age 8 in girls), precocious puberty has shown significantly rising incidence due to evolving dietary patterns and social environments., and is second only to childhood obesity. It occupies the second place among endocrine diseases in children and has become one of the common endocrine diseases in children.^[17]^ The occurrence of this disease is closely related to factors such as heredity, stimulation of external endocrine disruptors, exercise, and overnutrition. Children with precocious puberty develop early and complete the development process prematurely, causing the growth and development time to stop early, resulting in adult height lower than Children of the same age and gender are more likely to have behavioral problems such as social withdrawal, depression, and mental disorders, which affect their normal growth and development and are not conducive to their physical and mental health development.^[18,19]^ In the current treatment of children with precocious puberty, GnRH analogs are often given to regulate the endocrine system and control their growth and development process.^[20]^ At the same time, during the period of taking corresponding drug treatment, it is also necessary to carry out corresponding nursing work for the children and relieve abnormal emotions. Bundle nursing is a treatment and nursing measure based on the combination of evidence-based medicine concepts and clinical nursing work. It can implement targeted intervention measures for a certain disease and is conducive to providing patients with more optimized medical care services. It has achieved significant clinical results. effective.^[21]^ In this study, children with precocious puberty were given a cluster nursing combined with triptorelin treatment plan, which can effectively enhance their therapeutic effect, improve their quality of life, have good safety, and provide a reference for disease treatment.

Human reproductive function is regulated by the hypothalamus–pituitary–gonadal axis. The hypothalamus synthesizes and releases GnRH. The latter is released to the pituitary gland in the form of non-sustained nerve pulses and binds to the corresponding receptors on the surface of the pituitary cell membrane, thereby secreting LH., FSH, LH, and FSH will induce normal pubertal development and maturation mechanisms, promote height and weight growth, the manifestation of secondary sexual characteristics and development of internal/external genitalia^[22]^ characterizes precocious puberty, primarily caused by premature activation of the hypothalamus–pituitary–gonadal axis system., which promotes early secretion of GnRH and abnormal increases in LH and FSH levels, which in turn leads to the emergence of secondary sexual characteristics, accelerated bone development, and increased bone age. When the bone age exceeds the actual age, the epiphysis will be closed prematurely. This will then limit the increase in adulthood and cause adverse effects on children’s future life and psychology.^[23]^ GnRH analogues (GnRHa) are often recommended clinically as the primary therapeutic drug to suppress gonads, inhibit or slow down the development speed and degree of secondary sex characteristics, delay and inhibit abnormal bone age maturation induced by sex hormones, and prevent premature menstruation, premature epiphyseal closure, etc, to ensure normal growth and development of children.^[24]^ Wang et al^[25]^ reported that for the treatment of children with central precocious puberty, triptorelin outperforms leuprorelin in reducing pediatric E2 and FSH levels, enhancing ovarian function, and slowing bone age progression and growth velocity, yielding higher treatment effectiveness. Post-intervention results revealed a significantly greater total effective rate in the study group versus controls. While both groups showed elevated predicted adult height and reduced LH/T compared to pre-intervention levels, the study group maintained superior predicted adult height alongside significantly lower bone age, LH, and T than the control group.These outcomes align with prior findings, demonstrating that cluster nursing plus qurelin therapy effectively regulates physical growth in children with precocious puberty development and endocrine system of children, and improve clinical efficacy. Analysis of the reason may be that triptorelin, as a GnRH analogue, competitively inhibits GnRH secretion through hypothalamic analogues, promotes the secretion of gonadotropin from the anterior pituitary gland, reduces GnRH receptor sensitivity, and participates in and inhibits the pituitary gland. The gonadal axis system antagonizes a series of reactions caused by GnRH stimulation by the pituitary gland, reduces gonadal technical secretion, controls regional sex hormone levels in the ovary, uterus, testicles, etc, thereby inhibiting the development of secondary gonads, reducing LH and T levels, delaying bone development, and thus prolong the bone growth cycle and increase the expected adult height.^[26]^ The combined application of cluster nursing can provide targeted nursing measures and rationally control drug dosage according to the physical condition, weight and condition of children with precocious puberty, improve children’s compliance during treatment, follow medication on time and in the right amount, and maintain good physical condition., ensuring that the drug exerts its effect stably, thereby enhancing clinical efficacy.^[27]^

The treatment process of children’s precocious puberty is long, the diagnosis and treatment costs are high, and the economic burden is heavy, which will increase the social, economic and family burdens of children and their families. Because children’s minds are not fully developed, their treatment compliance, clinical efficacy and quality of life It completely depends on the attitude of the family and family members. At the same time, whether children with precocious puberty can have a sound personality and a healthy physical and mental state also depends on family, social and hospital care.^[28]^ Traditional nursing programs mostly focus on disease treatment, and the content and form of nursing staff work are too single, ignoring the real needs and psychological state of children.^[29]^ By establishing a professional nursing team, cluster care provides sustainable and effective nursing measures for children with precocious puberty based on their clinical characteristics, psychological status and other needs, helps children and their families improve their disease cognition, and provides them with targeted psychological support. Provide guidance, enhance their confidence in defeating the disease, encourage them to improve their compliance with drug treatment,strictly adhere to prescribed medication schedules. improve their mental state, nutritional status and physical fitness, strengthen the attention of patients and their families on disease prevention and control, and conscientiously implement various measures. This nursing work focuses on the quality of care and ensures the treatment and nursing effects of children with precocious puberty.^[30]^ This nursing method is usually combined with triptorelin therapy, and the medication is adjusted according to different body weights and physical conditions to ensure stable drug efficacy. In addition, the drug has a longer half-life and stronger affinity for GnRH receptors, which improves the overall therapeutic effect and improves the growth and development of children. condition, further improve the quality of life and reduce the incidence of adverse reactions. Post-intervention analysis demonstrated significant reductions in behavioral problem scores (depression, social withdrawal, hyperactivity, and aggression) across both cohorts compared to baseline values. The experimental group exhibited superior improvements in all behavioral domains versus controls. Additionally, quality of life measures encompassing family relationships, peer interactions, academic environment, living conditions, self-awareness, physical well-being, anxiety management, and mood regulation showed marked enhancement from pretreatment levels in both groups. The intervention cohort achieved significantly greater improvements across all quality of life dimensions compared to standard care recipients; conversely, adverse reaction incidence post-intervention was reduced in the study group.It is suggested that cluster nursing combined with triptorelin treatment can effectively reduce the behavioral problems of children with precocious puberty, improve patient QOL and decrease adverse event incidence.

Key limitations of this study include the restricted sample size and short follow-up period, which may impact the generalizability and long-term implications of the results. Additionally, potential confounding by unmeasured variables, such as variations in treatment adherence or individual psychological factors, may have influenced the outcomes. Furthermore, the cluster nursing plan has not yet become systematic, and the impact on long-term growth, development, and quality of life was not analyzed. Therefore, future studies with larger sample sizes and longer follow-up periods are needed to further assess the feasibility and broader applicability of the research plan.

Combined cluster nursing and triptorelin therapy yields effective improvement in clinical efficacy, inhibition of secondary sexual characteristic development, enhanced quality of life, high safety, and scientific basis for prevention/treatment in children with precocious puberty.

## Author contributions

**Conceptualization:** Hui Wang, Juan Chen.

**Data curation:** Hui Wang, Juan Chen.

**Formal analysis:** Hui Wang, Juan Chen.

**Investigation:** Hui Wang, Juan Chen.

**Methodology:** Hui Wang, Juan Chen.

**Validation:** Hui Wang, Juan Chen.

**Writing – original draft:** Hui Wang, Juan Chen.

**Writing – review & editing:** Hui Wang, Juan Chen.

## References

[1] Pereira A Busch AS Solares F Baier I Corvalan C Mericq V. Total and central adiposity are associated with age at gonadarche and incidence of precocious gonadarche in boys. J Clin Endocrinol Metab. 2021;106:1352–61. 33539513 10.1210/clinem/dgab064

[2] Jeong HR Lee HJ Shim YS Kang MJ Yang S Hwang IT. Inhibin B as a screening tool for early detection and treatment monitoring of central precocious puberty. Gynecol Endocrinol. 2020;36:768–71. 32162574 10.1080/09513590.2020.1718642

[3] Zurita-Cruz JN Villasís-Keever MA Manuel-Apolinar L. Altered cardiometabolic profile in girls with central precocious puberty and adipokines: a propensity score matching analysis. Cytokine. 2021;148:155660. 34334260 10.1016/j.cyto.2021.155660

[4] Fan T He J Wang Y Yu J Sun W. Generation of an induced pluripotent stem cell line (FDCHi006-A) from a 7-year-old girl with central precocious puberty. Stem Cell Res. 2021;56:102542. 34619645 10.1016/j.scr.2021.102542

[5] Vuralli D Gonc NE Ozon ZA Kandemir N Alikasifoglu A. Which parameters predict the beneficial effect of GnRHa treatment on height in girls with central precocious puberty? Clin Endocrinol (Oxf). 2021;94:804–10. 33460480 10.1111/cen.14420

[6] Gohil A Eugster EA. Gonadotropin-releasing hormone analogs for treatment of central precocious puberty in children younger than 2 years of age. J Pediatr. 2022;244:215–8. 34942182 10.1016/j.jpeds.2021.12.030

[7] Park HK Choo MS Shim YS. Adult height after gonadotropin-releasing hormone agonist treatment in girls with early puberty: a meta-analysis. Clin Endocrinol (Oxf). 2020;93:135–45. 32392622 10.1111/cen.14214

[8] Luo X Liang Y Hou L Wu W Ying Y Ye F. Long-term efficacy and safety of gonadotropin-releasing hormone analog treatment in children with idiopathic central precocious puberty: a systematic review and meta-analysis. Clin Endocrinol (Oxf). 2021;94:786–96. 33387371 10.1111/cen.14410 PMC8248422

[9] Coban OG Bedel A Onder A Adanir AS Tuhan H Parlak M. Psychiatric disorders, peer-victimization, and quality of life in girls with central precocious puberty. J Psychosom Res. 2021;143:110401. 33611071 10.1016/j.jpsychores.2021.110401

[10] Klein KO Freire A Gryngarten MG. Phase 3 trial of a small-volume subcutaneous 6-month duration leuprolide acetate treatment for central precocious puberty (vol 105, p 3660, 2020). J Clin Endocrinol Metab. 2020;105:e3660–71. 32738042 10.1210/clinem/dgaa479 PMC7442270

[11] Wannes S Elmaleh-Berges M Simon D. High prevalence of syndromic disorders in patients with non-isolated central precocious puberty. Eur J Endocrinol. 2019;179:373–80. 10.1530/EJE-18-0613 30324796

[12] Subspecialty Group of Endocrinologic, Hereditary and Metabolic Diseases, the Society of Pediatrics, Chinese Medical Association; Editorial Board, Chinese Journal of Pediatrics. Expert consensus on the diagnosis and treatment of central precocious puberty(2022). Zhonghua Er Ke Za Zhi. 2023;61:16–22. 36594116 10.3760/cma.j.cn112140-20220802-00693

[13] Chotipakornkul N Onsoi W Numsriskulrat N Aroonparkmongkol S Supornsilchai V Srilanchakon K. The utilization of basal luteinizing hormone in combination with the basal luteinizing hormone and follicle-stimulating hormone ratio as a diagnostic tool for central precocious puberty in girls. Ann Pediatr Endocrinol Metab. 2023;28:138–43. 37401058 10.6065/apem.2346072.036 PMC10329948

[14] Badawi N Fawaz L Amin A Kamel A Arafa N. The validity of the Bayley-Pinneau method in predicting final adult height at the onset of puberty in patients with classic congenital adrenal hyperplasia. Endokrynol Pol. 2021;72:301–7. 34010438 10.5603/EP.a2021.0039

[15] Lacalle Sisteré M Domènech Massons JM Granero Pérez R Ezpeleta Ascaso L. Validity of the DSM-oriented scales of the child behavior checklist and youth self-report. Psicothema. 2014;26:364–71. 25069556 10.7334/psicothema2013.342

[16] Qu S Wang P Wang M. A comparison of mood, quality of life and executive function among narcolepsy type 1 patients with or without ADHD symptoms in China. Sleep Med. 2022;97:47–54. 35717731 10.1016/j.sleep.2022.05.016

[17] Maione L Bouvattier C Kaiser UB. Central precocious puberty: recent advances in understanding the etiology and in the clinical approach. Clin Endocrinol (Oxf). 2021;95:542–55. 33797780 10.1111/cen.14475 PMC8586890

[18] Correa Brito L Rey RA. Taming idiopathic central precocious puberty: high frequency of imprinting disorders in familial forms. J Clin Endocrinol Metab. 2023;108:e636–7. 36794430 10.1210/clinem/dgad091 PMC10348455

[19] Chae HW Na JH Kwon A Kim H-S Lee Y-M. Central precocious puberty may be a manifestation of endocrine dysfunction in pediatric patients with mitochondrial disease. Eur J Pediatr. 2021;180:425–32. 32914201 10.1007/s00431-020-03804-3

[20] Ramos CO Canton APM Seraphim CE. Anthropometric, metabolic, and reproductive outcomes of patients with central precocious puberty treated with leuprorelin acetate 3-month depot (11.25 mg). J Pediatr Endocrinol Metab. 2021;34:1371–7. 34298591 10.1515/jpem-2021-0142

[21] Jin Q Zhou Y Yin D He H Liu Y Wu Y. Effects of cluster nursing on cardiac function and quality of life in coronary heart disease patients with chronic heart failure: a protocol of randomized controlled trial. Medicine (Baltim). 2022;101:e29091. 10.1097/MD.0000000000029091 PMC9276295 35446292

[22] Brito VN Canton APM Seraphim CE. The congenital and acquired mechanisms implicated in the etiology of central precocious puberty. Endocr Rev. 2023;44:193–221. 35930274 10.1210/endrev/bnac020 PMC9985412

[23] Zhou L Ren Y Li D. Timosaponin AIII attenuates precocious puberty in mice through downregulating the hypothalamic–pituitary–gonadal axis. Acta Biochim Pol. 2023;70:183–90. 36928746 10.18388/abp.2020_6450

[24] Popovic J Geffner ME Rogol AD. Gonadotropin-releasing hormone analog therapies for children with central precocious puberty in the United States. Front Pediatr. 2022;10:968485. 36268040 10.3389/fped.2022.968485 PMC9577333

[25] Wang L Jiang Q Wang M Xu J Jin J. The effect of triptorelin and leuprolide on the level of sex hormones in girls with central precocious puberty and its clinical efficacy analysis. Transl Pediatr. 2021;10:2307–12. 34733671 10.21037/tp-21-352 PMC8506057

[26] Yoo E Kim S Jung HL. Impact of 6-month triptorelin formulation on predicted adult height and basal gonadotropin levels in patients with central precocious puberty. Front Endocrinol (Lausanne). 2023;14:1134977. 36875449 10.3389/fendo.2023.1134977 PMC9982112

[27] Turan Miral M Hotun Sahin N. Psychosocial adaptation to precocious puberty: a nursing support program. J Child Adolesc Psychiatr Nurs. 2022;35:285–94. 35315171 10.1111/jcap.12376

[28] Kwon A Cho YI Kim HJ Suh J Kim DH. The mediating effects of parenting style on the relationship between parental stress and behavioral problems in girls with precocious puberty in Korea: a cross-sectional study. BMC Pediatr. 2023;23:352. 37438739 10.1186/s12887-023-04172-1 PMC10337196

[29] Park SJ Nam HR Choi EJ. Perception of precocious puberty among school-aged children in South Korea with the experience of treatment for precocious puberty: a Q methodological approach. Child Health Nurs Res. 2023;29:195–206. 37554087 10.4094/chnr.2023.29.3.195 PMC10415841

[30] Barkhordari-Sharifabad M Vaziri-Yazdi S Barkhordari-Sharifabad M. The effect of teaching puberty health concepts on the basis of a health belief model for improving perceived body image of female adolescents: a quasi-experimental study. BMC Public Health. 2020;20:370. 32197594 10.1186/s12889-020-08482-2 PMC7083033

